# One-month early time-restricted eating enhances cognition via white matter–cortical pathways in males with metabolic syndrome: evidence from TBSS and SBM analyses

**DOI:** 10.3389/fnut.2026.1753462

**Published:** 2026-03-13

**Authors:** Xin Jin, Linshuang Feng, Xiaoshi Li, Tingting Qu, Yunbing Wu, Zirui Wang, Keyao Hui, Hui Guo, Liwei Chen, Lei Wang, Yarong Wang

**Affiliations:** 1 Department of Radiology, The First Affiliated Hospital of Xi’an Jiaotong University, Xi’an, China; 2 Department of Radiology, Xi’an Daxing Hospital Affiliated to Yan’an University, Xi’an, China; 3 Xi’an Jiaotong University Health Science Center, Xi’an, China; 4 Department of Endocrinology and Metabolism, The First Affiliated Hospital of Xi’an Jiaotong University, Xi’an, China; 5 Fielding School of Public Health, University of California Los Angeles, Los Angeles, CA, United States

**Keywords:** dorsolateral prefrontal cortex, memory function, metabolic syndrome, surface-basedmorphometry, time-restricted eating, tract-based spatial statistics, white matter microstructure

## Abstract

**Background:**

Metabolic syndrome (MetS) is associated with white and gray matter structural abnormalities and cognitive decline, particularly in executive function and memory. Early time-restricted eating (eTRE) is a promising lifestyle intervention to improve metabolic and cognitive health, yet its effects on brain structure remain unclear.

**Objective:**

To investigate whether a 1-month eTRE intervention improves metabolic health, brain structure, and cognition in male MetS patients.

**Methods:**

Twenty-one males with MetS underwent a 1-month eTRE intervention. Diffusion tensor imaging (DTI), T1-weighted images, metabolic and cognitive measures including the Trail Making Test (TMT), Processing Speed (PS), and Rey Auditory Verbal Learning Test (RAVLT) were collected at baseline and post-intervention. White matter microstructure was analyzed using tract-based spatial statistics (TBSS), and cortical morphology was assessed using surface-based morphometry (SBM). Mediation analysis explored links between cognitive and structural changes.

**Results:**

After 1-month eTRE, body weight, BMI, fasting glucose, fasting insulin, and HOMA-IR significantly decreased, while QUICKI increased (all *p* < 0.01). Cognitive performance improved in memory, PS, and executive function. TBSS revealed fractional anisotropy (FA) increases in the left anterior thalamic radiation (ATR_L) and bilateral corticospinal tracts. SBM showed cortical thinning in the right dorsolateral prefrontal cortex (DLPFC) associated with improved delayed recall, TMT performance and PS. Mediation analysis demonstrated that the right DLPFC thickness significantly mediated the relationship between the ATR_L FA and delayed recall (ACME = 10.07, 95% CI = 1.10–37.84, *p* = 0.021).

**Conclusion:**

One month of eTRE was associated with metabolic improvement and coordinated changes in brain structure and cognition in males with MetS, consistent with adaptive thalamo–prefrontal network remodeling.

## Introduction

Metabolic syndrome (MetS) is a cluster of risk factors including abdominal obesity, hyperglycemia, dyslipidemia, and hypertension, with rising prevalence worldwide, particularly among middle-aged and elderly populations (1). Epidemiological studies in China indicate that more than 19% of adults are affected (2). Men are particularly susceptible to cardiometabolic disturbances (3), which may accelerate cognitive decline and brain structural changes (4). Metabolic and brain alterations during young-to-middle adulthood remain partially modifiable, representing a critical window for early intervention (5, 6). These findings underscore the need for preventive strategies in high-risk populations.

MetS is closely associated with widespread brain structural alterations and cognitive decline. Core pathophysiological mechanisms involve insulin resistance (7–9), vascular dysfunction (10, 11), inflammation, and oxidative stress (12), which converge to disrupt neuronal energy metabolism (13, 14), damage myelin and oligodendrocytes, and reduce cerebral blood flow, ultimately accelerating white matter degeneration (15, 16) and cortical alterations. As these metabolic and vascular insults accumulate, individuals with MetS frequently exhibit cognitive difficulties (17, 18), with the most consistently affected domains being memory, executive function, and processing speed (19, 20).

White matter abnormalities represent one of the most consistent neuroimaging findings in MetS. Diffusion tensor imaging (DTI) studies have shown widespread reductions in fractional anisotropy (FA) across major long-range tracts—including the anterior thalamic radiation, superior longitudinal fasciculus, and corpus callosum (21–24) indicating diffuse axonal and myelin microstructural damage. Because these pathways link the thalamus with prefrontal and parietal association cortices, their disruption undermines the structural foundation of cognitive control networks. Besides, individuals with MetS often exhibit slowed processing speed, impaired attentional regulation, and reduced executive flexibility, reflecting dysfunction within thalamo–prefrontal and frontoparietal systems (25, 26).

MetS is also accompanied by notable gray matter alterations. Structural reductions in the prefrontal cortex, hippocampus, and temporal regions (27, 28), affect key hubs responsible for memory, goal-directed behavior, and cognitive regulation. The prefrontal cortex supports planning, organization, and information manipulation (29), whereas the hippocampus is central to episodic memory encoding and retrieval (30). When these cortical systems are compromised, integrative functions such as memory consolidation, cognitive flexibility, and complex decision-making are likely impaired. Overall, converging evidence indicates that MetS disrupts both white matter and gray matter pathways, which together form the structural basis of higher-order cognition.

Lifestyle modification is a promising strategy to mitigate MetS-related complications (31, 32). Early time-restricted eating (eTRE), an intermittent fasting regimen restricting food intake to a daily window of 6–8 hours (33, 34), improves insulin sensitivity, lipid metabolism, and inflammatory status (35, 36), and has better compliance than exercise-based interventions. Recent evidence from animal models (37, 38) and small clinical trials (39, 40) suggests that TRE may also exert neuroprotective effects by reducing neuroinflammation and metabolic stress, thereby promoting myelin preservation and cortical stability. These changes may enhance brain network integration that underlies executive function and memory, potentially alleviating white matter and gray matter injury as well as cognitive decline associated with MetS (41, 42).

In this study, we examined the effects of a 1-month eTRE intervention in male MetS patients. We assessed changes in metabolic and cognitive outcomes together with brain structural alterations (43–45) focusing on white matter and gray matter by tract-based spatial statistics (TBSS) and surface-based morphometry (SBM) (46, 47). We used mediation analysis to explore the relationship between cognitive improvements and structural changes. We hypothesized that eTRE would improve metabolic health, cognitive function, and brain structure, and that white matter and cortical features might be interrelated and jointly influence cognitive outcomes.

## Materials and methods

### Participants

This study enrolled 21 males with MetS (aged 26–51 years), recruited from the Endocrinology Outpatient Department of Xi’an Daxing Hospital, hospital announcements, and online advertisements. Written informed consent was obtained from all participants before enrollment. The study was conducted in accordance with the Declaration of Helsinki and approved by the Medical Ethics Committee of Xi’an Daxing Hospital (Approval Number: KY2024-025). Inclusion and exclusion criteria were as follows (48).

### Inclusion criteria

(1) Age: from 25 to 55 years.
(2) No eating disorder.
(3) No severe mental illnesses (e.g., schizophrenia, bipolar disorder, major depressive disorder, severe anxiety disorders, or other severe psychotic disorders).
(4) Voluntarily sign the informed consent form.
(5) Maintaining stable weight (weight change <10%) in the 3 months prior to the study.
(6) If participants are taking hypoglycemic, antihypertensive, lipid-lowering, and cardiovascular medications, they are not allowed to adjust the dose during the intervention period.
(7) Clinically diagnosed with MetS (49), with at least 3 of the following 5 abnormalities: (no need to exclude those already diagnosed with hypertension/diabetes);

- Waist circumference for men ≥90 cm, for women ≥85 cm;
- Fasting triglycerides ≥1.7 mmol/L;
- Fasting high-density lipoprotein cholesterol <1.04 mmol/L;
- Systolic/diastolic blood pressure ≥130/85 mmHg, and (or) those who have been diagnosed with hypertension and are being treated;
- Fasting blood glucose ≥6.1 mmol/L and (or) 2-h postprandial blood glucose ≥7.8 mmol/L, and (or) those who have been diagnosed with diabetes and are being treated.

### Exclusion criteria

(1) Night shift workers.
(2) History of weight loss surgery.
(3) MRI examination contraindications.
(4) Presence of neurological disorders or brain dysfunction.
(5) History of head injury or brain lesions.
(6) Addiction to tobacco or alcohol.
(7) Exclusion related to safety concerns.
(8) Inability to adhere strictly to the study protocol.
(9) History of endocrine disorders or obesity resulting from single-gene. Mutations, including but not limited to hypothalamic obesity, pituitary obesity, hypothyroid obesity, Cushing’s syndrome, insulinoma, acromegaly and hypogonadism.
(10) Experienced two or more hypoglycemic events in the 6 months before screening, defined as blood glucose <2.8 mmol/L, or blood glucose not reaching <2.8 mmol/L but with evident hypoglycemic symptoms.
(11) History of significant illness or related diseases, such as inflammatory diseases, rheumatic autoimmune diseases, adrenal diseases, malignant tumors, type 1 diabetes, liver cirrhosis, chronic kidney disease, acquired immunodeficiency syndrome, eating disorders, mental disorders, and adverse cardiovascular events.
(12) Severe gastrointestinal diseases or gastrointestinal surgeries within the past 12 months, actively participating in weight loss programs, using medications that affect weight or energy balance, currently participating in other weight management programs, or receiving prescribed diets due to specific diseases or taking any medications that affect appetite.

### Study design

This study adopted a 1-month within-subject pre-post design to examine the effects of eTRE in participants with MetS. The study sample included only male participants. Participants were instructed to restrict their daily eating window to 8 h (08:00–16:00). Within this window, they were allowed to eat ad libitum without calorie counting or food-type restrictions, but were advised to maintain their habitual dietary patterns. A balanced and varied diet was encouraged, ensuring adequate intake of protein, carbohydrates, fats, vitamins, and minerals, while avoiding excessive consumption of high-calorie, high-fat, and high-sugar foods. During the remaining 16-h fasting period, participants were encouraged to drink water freely. Consumption of any foods, tea, coffee, or other caloric beverages was not permitted. The study emphasized participants’ comfort and safety, reminding them to listen to their bodies and report any discomfort or adverse reactions during the fasting period. All outcomes, including cognitive tests, biochemical parameters, and neuroimaging measures, were collected at baseline and after 1-month of intervention.

### Intervention and adherence management

To monitor all participants during the fasting window, participants used a Continuous Glucose Monitor (CGM, from WeiTai Health Medical Technology Co., LTD China Shanghai), which was connected to their mobile phones and the endocrinologist phones by an APP for real-time monitoring. If any participant ate outside the fasting window, the endocrinologist would receive alerts indicating elevated blood glucose levels, and immediately Contact the participants keep to follow the dietary rules.

A separate social media group was established for each participant, consisting of the participants, one endocrinologist, one nutrition nurse, and three radiologists. Physicians provided daily supervision and encouragement within the group, while participants were required to upload photos of their meals each day. All physicians in the group responded to participants’ questions in real time, offering continuous support and motivation to enhance adherence and confidence in the intervention.

Comprehensive data quality control and adherence verification procedures were implemented throughout the study. Detailed descriptions of the CGM configuration, spike detection algorithm, calibration procedures, and adherence feedback mechanisms are provided in the Supplementary Methods.

### Clinical and cognitive assessments

At baseline, demographic and clinical characteristics were collected, including age, weight, height, education, and smoking and drinking history. Body weight and height were measured using the InBody 770 body composition analyzer (InBody, Seoul, Korea), with a precision of ±0.1 kg for weight and ±0.5 cm for height. Measurements were taken barefoot and in light clothing. Body mass index (BMI) was calculated as weight (kg)/height^2^(m^2^), and assessed at both baseline and post-intervention. Venous blood samples were obtained at the Clinical Laboratory of Xi’an Daxing Hospital between 07:40 and 09:00 following an overnight fast (from 20:00 the previous evening), both at baseline and 1-month eTRE. Laboratory assays included triglycerides (TG), total cholesterol (TC), high-density lipoprotein cholesterol (HDL-C), low-density lipoprotein cholesterol (LDL-C), fasting plasma glucose (FPG), fasting insulin (FINS), and C-reactive protein. Insulin resistance and sensitivity indices were calculated as follows (the homeostatic model assessment of insulin resistance [HOMA-IR] and the quantitative insulin sensitivity check index [QUICKI] formulas use FPG in mmol/L and FINS in μIU/mL, the results are dimensionless):


$$\text{HOMA}-\mathrm{IR}=\frac{\mathrm{FPG}\times \text{FINS}}{22.5\times 1.7}$$

$$\text{QUICKI}=\frac{1}{\text{logFPG}+\log(\text{FINS}/1.7)}$$

Psychiatric status was screened at baseline using clinical history and standardized questionnaires to rule out severe mental illnesses, including the Hamilton Anxiety Scale, Beck Depression Inventory, Patient Health Questionnaire-9, and the 12-Item Short Form Health Survey, which assesses overall mental and physical health status. Participants with a prior DSM-5–based diagnosis of severe psychiatric disorders as determined from clinical history were excluded.

Cognitive assessments were measured with Trail Making Test (TMT), Processing Speed (PS) and Rey Auditory Verbal Learning Test (RAVLT) at baseline and 1-month eTRE.

### Image acquisition

MRI data were acquired using a 3.0 T MRI system (MAGNETOM Prisma, Siemens Healthineers, Erlangen, Germany) with a 32-channel coil. High-resolution T1-weighted anatomical images were obtained using a 3D MPRAGE sequence with the following parameters: scan time = 3min 9s, number of slices = 176, voxel size = 1.0 mm × 1.0 mm × 1.0 mm, repetition time (TR) = 1540ms, echo time (TE) = 2.99ms, flip angle = 9°, field of view (FOV) = 224 mm × 224 mm, and image resolution = 224 × 224. The DTI scan was performed using the following parameters: total scan duration = 10 min 42 s, slices = 64, voxel size = 2.0 mm × 2.0 mm × 2.0 mm, TR = 3800ms, TE = 92ms, flip angle = 52°, FOV = 220 mm × 220 mm, and matrix size = 110 × 110. Two *b*-values were acquired: *b* = 0 s/mm^2^ (with both anterior–posterior and posterior–anterior phase-encoding directions) and *b* = 1,000 s/mm^2^ (posterior–anterior, 30 diffusion directions).

### DTI preprocessing and TBSS analysis

Diffusion-weighted data in DICOM format were first converted to NIfTI using MRICroGL (50). Preprocessing was performed with FSL (51) (version 6.0.7.16). Images were corrected for eddy current–induced distortions and motion using *topup* and *eddy*. Brain extraction was conducted with the Brain Extraction Tool (BET) (52). Diffusion tensors were fitted to the eddy-corrected data, and white matter microstructure parameter maps including FA, axial diffusivity (AD), mean diffusivity (MD), and radial diffusivity (RD) were generated using the FSL Diffusion Toolbox.

All white matter microstructure parameter maps were conducted using the TBSS pipeline (53). The parameter maps of each participant were nonlinearly registered to the MNI152 1 × 1 × 1 mm^3^ standard space, and a mean white matter skeleton was created to represent the central white matter tracts common to the group. Individual parameter maps were projected onto this skeleton for voxel-wise statistical analysis.

Group comparisons between 1-month eTRE and baseline were assessed using non-parametric permutation testing (5,000 permutations) with a paired t-test design, combined with threshold-free cluster enhancement (TFCE) and family-wise error (FWE) correction (*p* < 0.05). Significant clusters were anatomically localized using the JHU White-Matter Tractography Atlas (maxprob version). Each voxel within significant clusters was assigned to the tract with the highest probability in the atlas for qualitative tract identification.

### T1 preprocessing and SBM analysis

High-resolution T1-weighted images were processed using the Computational Anatomy Toolbox (CAT12, version 12.8; https://neuro-jena.github.io/cat/) implemented in SPM12 (Statistical Parametric Mapping, Wellcome Centre for Human Neuroimaging, London, UK; https://www.fil.ion.ucl.ac.uk/spm/). Images were corrected for bias-field inhomogeneities and segmented into gray matter, white matter, and cerebrospinal fluid. Cortical surfaces were reconstructed and topologically corrected.

Cortical thickness was calculated as the distance between the white matter and pial surfaces, and smoothed with a 12-mm Full Width at Half Maximum (FWHM) Gaussian kernel. Sulcal depth was estimated from the standardized Euclidean distance between the central cortical surface and its convex hull, and fractal dimension was calculated using spherical harmonic reconstructions; sulcal depth and fractal dimension were smoothed with a 20-mm FWHM Gaussian kernel.

All individual surface measures were registered to MNI space using surface-based normalization. Vertex-wise comparisons of cortical thickness, sulcal depth, and fractal dimension between baseline and post-intervention were performed using paired *t*-tests. Anatomical labeling of significant clusters was performed using the Destrieux 2009 atlas in CAT12. Multiple comparisons were corrected using the FDR at *p* < 0.05.

### Statistical analysis

Clinical, metabolic and cognitive data were analyzed using SPSS (version 26.0, IBM Corp., Armonk, NY, USA). Normality was assessed with the Shapiro–Wilk test. Normally distributed data were compared between baseline and post-intervention using paired *t*-tests, and non-normally distributed data were analyzed with the Wilcoxon signed-rank test. Continuous variables are presented as mean ± standard deviation (SD). A two-tailed *p* < 0.05 was considered statistically significant.

Correlation analysis was performed to assess the relationship between changes in white matter microstructure parameters, cortical morphology measures, clinical, metabolic, and cognitive outcomes (including TMT, PS, and RAVLT). Pearson correlation was used for normally distributed data, and Spearman’s rank correlation was used for non-normally distributed data.

To explore the relationship between cognitive improvements and structural changes, brain regions showing significant changes were selected and mediation analysis was performed using R (version 4.4.1) with the “mediation” package (54). The significance of indirect effects was assessed using a bootstrapping-based resampling approach with 5,000 resamples. Significance was set at *p* < 0.05 (two-tailed).

## Results

### Demographic and clinical measurements

A total of 21 males with MetS (mean age 35.67 ± 5.91 years), completed the 1-month eTRE intervention. Only one participant reported regular alcohol consumption, with an average daily intake of approximately 0.8 standard drinks. Baseline demographic and clinical characteristics are presented in Table 1. After 1-month of eTRE, participants showed significant reductions in body weight and BMI (*p* < 0.001). In terms of metabolic risk factors, TC, FPG, fasting insulin, and HOMA-IR significantly decreased, while QUICKI significantly increased (*p* < 0.01). TG, HDL-C, LDL-C, and hs-CRP showed a tendency toward improvement, but the changes did not reach statistical significance. Cognitive assessments demonstrated significant improvements in memory, processing speed, and executive function. Immediate recall and delayed recall scores on the RAVLT were significantly higher after the intervention (*p* < 0.01). Processing speed (PS) improved, as indicated by a shorter PS average reaction time (*p* = 0.002). Executive function also improved: the total completion time for the TMT was significantly reduced (*p* = 0.035). Although TMT-A and TMT-B completion times both decreased after the intervention, only the reduction in TMT-B reached statistical significance (TMT-A: *p* = 0.131; TMT-B: *p* = 0.032). TMT ΔB–A and the TMT-B/A Ratio also showed reductions, but these changes did not reach statistical significance. (Table 1).

**Table 1:** Demographic characteristics and changes in metabolic risk factors, inflammatory markers and cognitive performance after 1-month eTRE intervention in MetS patients (*n* = 21).

Variable	Before eTRE	After eTRE	*t*-value	*p*-value
Demographic characteristics				
Age (years)	35.67 ± 5.91	NA	NA	NA
Education (years)	15.48 ± 1.03	NA	NA	NA
Height (cm)	174.52 ± 7.16	NA	NA	NA
Smoking, *n* (%)	6 (28.5)	NA	NA	NA
Cigarettes/day (smokers only)	11.3 ± 14.5	NA	NA	NA
Weight (kg)	96.11 ± 13.44	91.67 ± 12.44	−8.44	<0.001***
BMI (kg/m^2^)	31.58 ± 4.34	30.12 ± 4.06	−8.54	<0.001***
Metabolic risk factors				
TG (mmol/L)	2.96 ± 2.06	2.11 ± 1.47	−1.76	0.094
TC (mmol/L)	4.89 ± 1.19	4.48 ± 0.86	−2.21	0.039*
HDL-C (mmol/L)	1.09 ± 0.73	0.93 ± 0.16	−1.02	0.320
LDL-C (mmol/L)	2.71 ± 0.81	2.74 ± 0.73	0.21	0.838
FPG (mmol/L)	6.24 ± 1.41	5.77 ± 0.91	−2.18	0.042*
FINS (μIU/mL)	21.68 ± 15.80	15.10 ± 9.98	−3.31	0.004*
HOMA-IR	5.83 ± 4.00	3.87 ± 2.48	−3.53	0.002*
QUICKI	0.30 ± 0.02	0.33 ± 0.03	3.71	0.001*
Inflammatory markers				
hs-CRP (mg/L)	4.11 ± 3.36	3.35 ± 2.38	−1.25	0.226
RAVLT (scores)				
Immediate recall (trials 1–5)	45.71 ± 7.53	54.43 ± 9.72	4.75	<0.001***
Interference recall	5.56 ± 2.01	5.74 ± 1.71	−0.33	0.740
Delayed recall	9.48 ± 2.91	11.33 ± 2.80	3.47	0.002*
PS average reaction time (s)	1.20 ± 0.27	1.07 ± 0.23	−3.52	0.002*
TMT test				
TMT (sec)	198.27 ± 59.32	177.90 ± 51.28	−2.26	0.035*
TMT-A	60.12 ± 22.61	54.29 ± 16.41	−1.57	0.131
TMT-B	138.14 ± 44.64	120.52 ± 38.39	−2.31	0.032*
TMT delta B-A (sec)	78.02 ± 38.59	66.24 ± 30.20	−1.44	0.165
TMTB/A ratio	2.46 ± 0.78	2.25 ± 0.52	−1.07	0.297

Continuous variables are presented as mean ± SD [range]. BMI, body mass index; TG, triglycerides; TC, total cholesterol; HDL-C, high-density lipoprotein cholesterol; LDL-C, low-density lipoprotein cholesterol; FPG, fasting plasma glucose; FINS, fasting insulin; HOMA-IR, homeostatic model assessment of insulin resistance; QUICKI, quantitative insulin sensitivity check index; hs-CRP, high-sensitivity C-reactive protein; RAVLT, rey auditory verbal learning test; PS, processing speed; TMT, trail making test; TMT delta B–A, TMT Part B minus Part A; TMT B/A ratio, ratio of TMT-B to TMT-A.

### TBSS analysis

**Figure 1:**
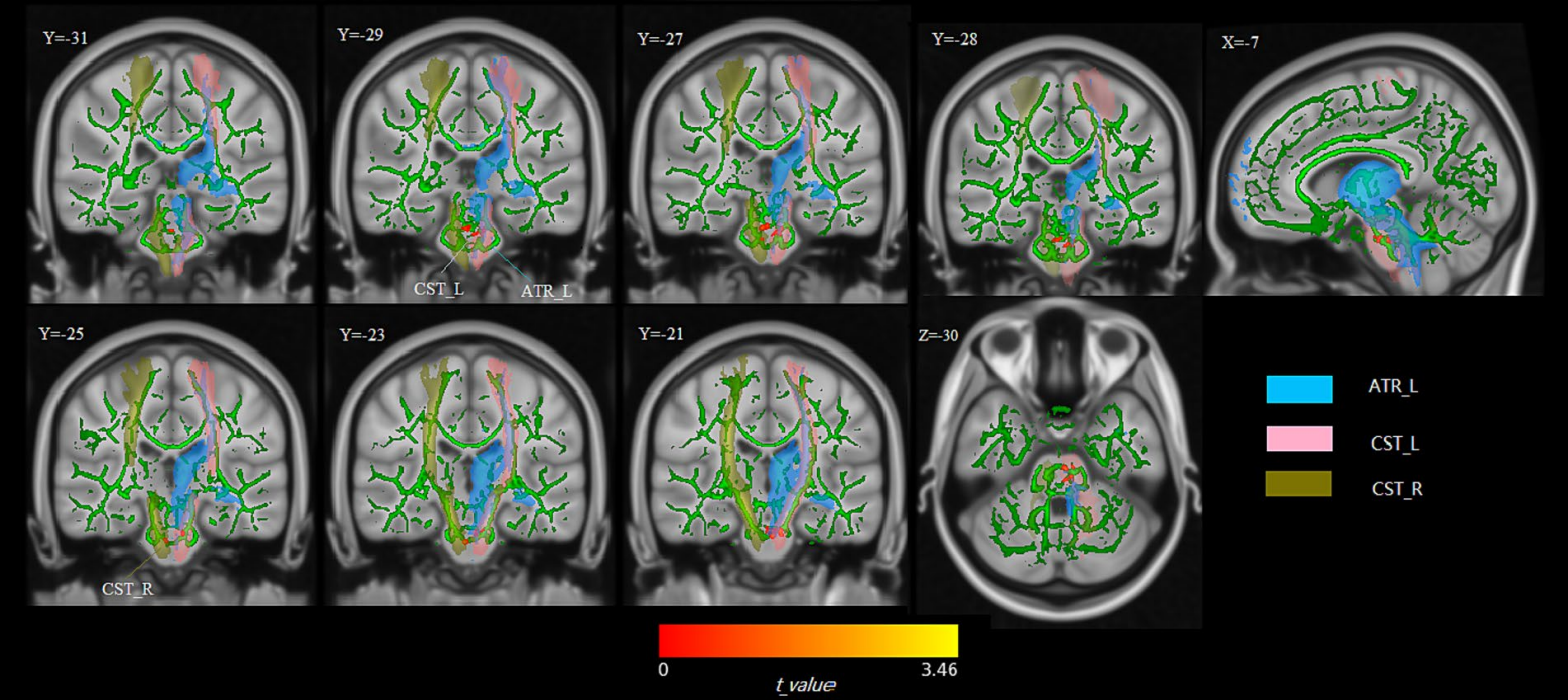
White matter regions showing significantly increased FA after the 1-month eTRE intervention compared to baseline in male MetS patients (*n* = 21). Significantly increased FA was observed in clusters located in the left anterior thalamic radiation (ATR_L) and bilateral corticospinal tracts compared to baseline (*p* < 0.05, FWE corrected with TFCE). Significant FA results are displayed after being thickened for visualization using the FSL tbss_fill script. The significant clusters (in red) indicate regions of FA increase overlapping with predefined tract masks: the light-blue mask represents ATR_L, the pink mask represents CST_L, and the brown mask represents CST_R. All masks were defined using the JHU white-matter tractography atlas, probabilistic without thresholding, included in FSL v6.0.7.16. Brain maps are shown in standard MNI152 space.

Compared to baseline, significant increases in FA were observed in clusters located in the ATR_L and bilateral corticospinal tracts after the 1-month eTRE intervention (*p* < 0.05, FWE corrected with TFCE). No significant changes were observed in other parameters, including AD, MD, and RD (*p* > 0.05) (Figure 1). Detailed results are presented in Supplementary Table S1.

### SBM analysis

**Figure 2:**
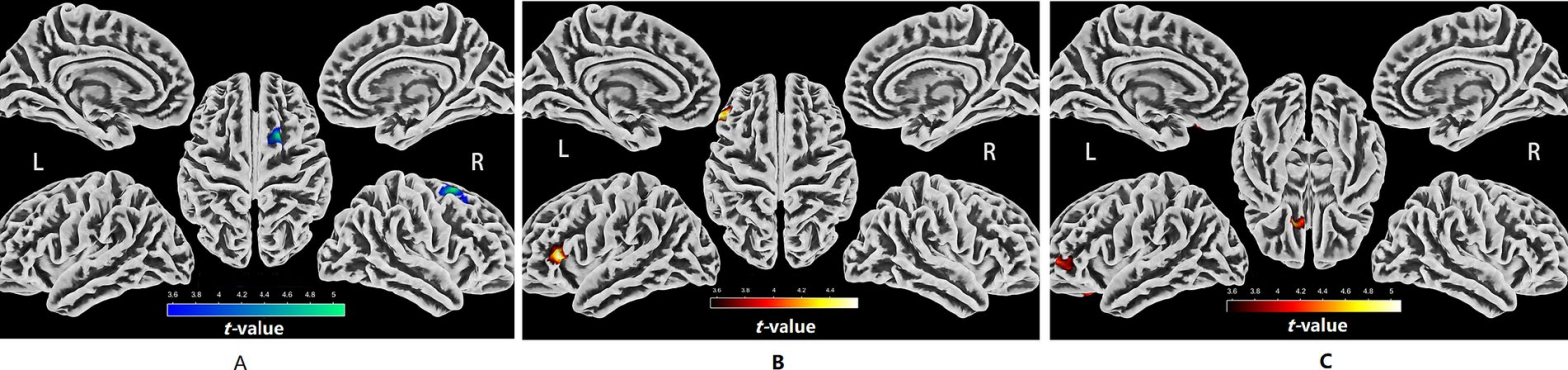
Cortical regions showing significant surface-based morphometric changes after the 1-month eTRE intervention in male patients with MetS (*n* = 21). Compared with baseline, significant reductions in cortical thickness were observed in a cluster located in the right DLPFC **(A)**, while significant increases in sulcal depth were found in a cluster spanning in the left inferior frontal sulcus and the left inferior frontal gyrus, triangular part **(B)**. In addition, fractal dimension significantly increased in clusters located in the left DLPFC and OFC **(C)** (all *p* < 0.05, FDR corrected).

Cortical thickness decreased significantly in a cluster located in the right dorsolateral prefrontal cortex (DLPFC) after the 1-month eTRE intervention compared to baseline (Figure 2A). Sulcal depth significantly increased in a cluster spanning the left inferior frontal sulcus and the left inferior frontal gyrus, triangular part (Figure 2B). In addition, fractal dimension significantly increased in clusters located in the left DLPFC and orbitofrontal cortex (OFC) (Figure 2C). All reported results were significant after FDR correction (*p* < 0.05). Detailed results for SBM analysis are presented in Supplementary Tables S2a,b,c.

### Correlation analysis

Increased FA in the ATR_L cluster was significantly correlated with metabolic improvements including reduced fasting glucose (*r* = −0.513, *p* = 0.017), increased QUICKI (*r* = 0.492, *p* = 0.024). The corresponding correlation scatter plots are provided in the Supplementary Figure S1.

The reduction in cortical thickness in the right DLPFC cluster was significantly associated with improved cognitive performance, including better delayed recall (*r* = −0.505, *p* = 0.020), lower TMT B/A Ratio (*r* = −0.463, *p* = 0.035), and faster processing speed (*r* = 0.520, *p* = 0.016). The corresponding correlation scatter plots are provided in the Supplementary Figure S2.

### Mediation analysis

**Figure 3:**
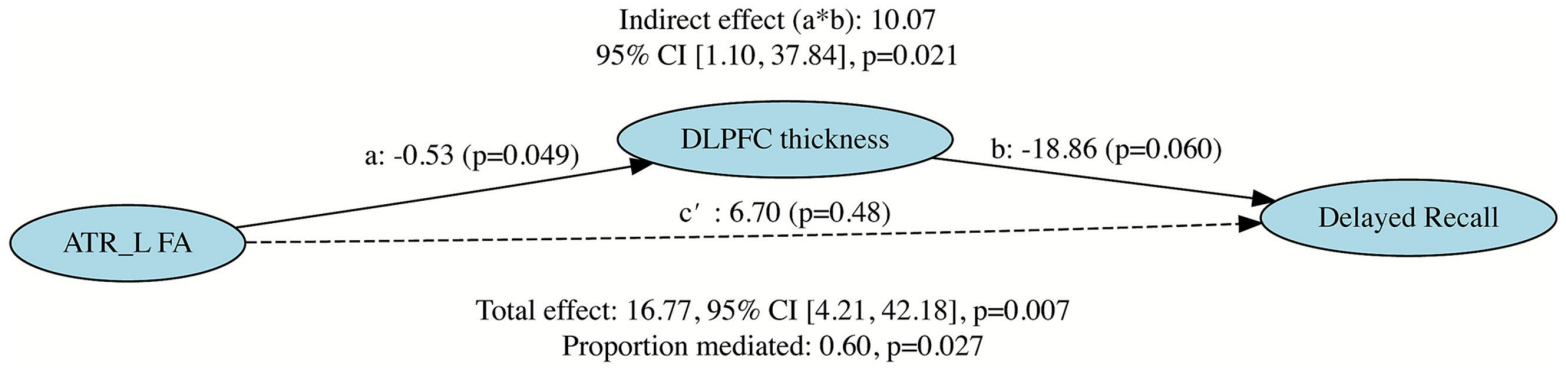
Mediation model examining the role of cortical thickness in the right DLPFC cluster in the relationship between FA in the ATR_L cluster and delayed recall performance. Path coefficients are unstandardized. The indirect effect (a × b) was tested using bootstrap procedures with 5000 resamples. *p < 0.05, **p < 0.01. The analysis showed a significant indirect effect (ACME = 10.07, 95% CI = 1.10–37.84, *p* = 0.021), with cortical thickness in the right DLPFC cluster mediating approximately 60% of the total effect between FA in the ATR_L cluster and delayed recall (95% CI = 0.07–2.59, *p* = 0.027).

Following the 1-month eTRE intervention, significant correlations were observed between delayed recall and increased FA in the ATR_L cluster and reduced cortical thickness in the right DLPFC cluster. To explore the relationship between cognitive improvements and structural changes, a mediation analysis was conducted with FA in the ATR_L cluster as the independent variable (X), cortical thickness in the right DLPFC cluster as the mediator (M), and delayed recall performance as the dependent variable (Y). The analysis revealed a significant indirect effect (ACME = 10.07, 95% CI = 1.10–37.84, *p* = 0.021), indicating that reduced cortical thickness in the right DLPFC cluster significantly mediated the relationship between FA in the ATR_L cluster and delayed recall. The direct effect of FA in the ATR_L cluster on delayed recall was nonsignificant (ADE = 6.70, 95% CI = −17.96–27.09, *p* = 0.48). The total effect was significant (16.77, 95% CI = 4.21–42.18, *p* = 0.007), with approximately 60% of the effect mediated through cortical thickness in the right DLPFC cluster (95% CI = 0.07–2.59, *p* = 0.027). The individual paths showed that FA in the ATR_L cluster was marginally significantly associated with cortical thickness in the right DLPFC cluster (path a: *β* = −0.53, *p* = 0.049), and cortical thickness in the right DLPFC cluster was marginally significantly associated with delayed recall performance (path b: *β* = −18.86, *p* = 0.060). The negative coefficients for both paths a and b resulted in a positive indirect effect, consistent with a suppression effect. The marginal significance of individual paths may reflect the modest sample size (*n* = 21), but the robust bootstrap test of the indirect effect provides confidence in the mediation pattern (Figure 3).

## Discussion

This study demonstrates that a 1-month eTRE intervention significantly improved metabolic health, white-matter integrity, cortical morphology, and cognitive performance—particularly executive function and memory in male patients with MetS. Using TBSS and SBM, we provide converging evidence for the neuroprotective effects of eTRE, linking structural remodeling with metabolic and cognitive enhancement. These findings are consistent with a mechanistic model in which improved metabolic regulation, achieved through enhanced insulin sensitivity, and stabilized glucose availability, may promote structural plasticity across thalamo-cortical networks. Strengthening of the ATR_L may enhance information flow to the ipsilateral prefrontal cortex, while cortical refinement occurs in a hemispherically differentiated manner: the right DLPFC exhibits cortical thinning, whereas the left DLPFC shows increased fractal dimension along with OFC involvement. These hemispherically distinct changes are likely coordinated across the bilateral prefrontal network, reflecting functional integration between left and right prefrontal regions to optimize executive and memory-related processing (55). This coordinated remodeling between white and gray matter provides a neurobiological basis for the observed cognitive gains and highlights the DLPFC as a critical hub potentially involved in the beneficial effects of eTRE on brain structural remodeling and cognitive function in MetS (56, 57).

The improvements in clinical, cognitive, and metabolic measures observed in this study are consistent with previous evidence (40). After 1-month of eTRE, body weight, BMI, fasting glucose, fasting insulin, and HOMA-IR significantly decreased, while QUICKI increased, indicating enhanced insulin sensitivity and metabolic flexibility (58). Enhanced insulin signaling is crucial for glucose uptake and energy metabolism in the brain, particularly in the prefrontal cortex and hippocampus (29, 30), regions essential for memory and executive functions. Improved insulin sensitivity may therefore promote neuronal activity, synaptic plasticity, and cognitive performance. Although most lipid parameters and hs-CRP did not reach statistical significance, with TC showing only a small statistically significant decrease (59), the observed cognitive enhancements especially in delayed recall, processing speed, and executive function support the idea that even short-term metabolic improvements can enhance brain function (60). Complex executive processes, such as those measured by the TMT-B/A ratio, may require longer interventions or more extensive structural reorganization to manifest.

TBSS revealed increased FA in the ATR_L and bilateral corticospinal tracts after eTRE, indicating enhanced white-matter integrity (61). The ATR_L serves as a key thalamo-cortical pathway projecting to the ipsilateral prefrontal cortex, and strengthening of this tract likely reflects improved axonal organization and myelin coherence. Given that insulin resistance (39), inflammation, and oxidative stress are known to impair white matter microstructure (13), the observed increase in FA following eTRE may reflect improved metabolic regulation that supports healthier axonal and myelin organization. Enhanced integrity of the ATR_L may facilitate more efficient thalamo-prefrontal communication and contribute to coordinated interactions between left and right prefrontal regions, supporting higher-order cognitive operations such as attention, working memory, and executive control. Consistent with this interpretation, increases in ATR_L FA were associated with better metabolic profiles, including reduced fasting glucose and improved insulin sensitivity, suggesting a potential link between systemic metabolic regulation and central structural remodeling.

Beyond white matter, cortical surface analyses revealed that eTRE induced cortical thinning in the right DLPFC (13, 21), accompanied by improved cognitive performance (62). While cortical thinning is often interpreted as atrophy, accumulating neuroimaging evidence suggests that modest thinning within association cortices can also reflect functional optimization (63). Under conditions of improved metabolic homeostasis, synaptic pruning and dendritic remodeling may eliminate redundant or inefficient connections, yielding a leaner yet more efficient cortical network (64). Thus, the cortical thinning observed in the DLPFC likely represents adaptive neural reorganization that enhances signal processing efficiency and functional connectivity, rather than degenerative loss. In contrast, increased fractal dimension in the left DLPFC and orbitofrontal cortex suggests complementary structural refinement that may enhance functional integration across prefrontal regions, supporting coordinated executive and memory processes (21). Together, these hemispherically differentiated patterns likely reflect adaptive neural reorganization rather than degenerative change, consistent with the observed improvements in processing speed, executive function, and memory.

The coupling between DLPFC morphology and ATR_L integrity provides important insight into how eTRE may enhance cognition. As a thalamo–prefrontal pathway, the ATR conveys dense thalamic input to the DLPFC and plays a key role in executive control, working memory, and goal-directed behavior. Recent human imaging and electrophysiological studies show that thalamic activity dynamically modulates prefrontal computations during cognitive control tasks, while structural connectivity along the thalamo–prefrontal axis is closely associated with individual differences in executive ability and processing speed (26, 65). The DLPFC serves as a hub integrating information from thalamic and parietal regions within the fronto-thalamo–parietal control network, coordinating top-down regulation, attentional shifting, and performance monitoring (25). Enhanced synchronization within this circuit may therefore improve the efficiency of cognitive control and neural transmission, providing a neural substrate for the observed behavioral improvements. Moreover, the metabolic enhancement induced by eTRE, reflected in improved insulin sensitivity, may help maintain axonal integrity and synaptic efficiency within these long-range pathways, consistent with our finding that greater ATR_L integrity and DLPFC remodeling were associated with better executive performance and faster processing (66).

Regarding memory, the eTRE intervention improved overall memory performance. Short-term memory (immediate recall) is supported primarily by transient neural activation within the frontoparietal network (67), whereas long-term memory (delayed recall) depends on more stable synaptic plasticity and coordinated interactions across thalamo-prefrontal-hippocampal circuits (68). The ATR conveys projections from the anterior and midline thalamic nuclei (69), which are critical for memory consolidation and retrieval. The DLPFC supports strategic retrieval, organizational processing, and top-down control during delayed recall (70).

Our mediation analysis suggested that DLPFC remodeling mediated the relationship between ATR_L integrity and delayed recall. Enhanced ATR_L integrity may support more reliable thalamo–prefrontal communication within ipsilateral pathways, which contributes to coordinated activity across bilateral prefrontal regions during long-term memory retrieval (71). Such bilateral coordination may be facilitated by interhemispheric communication through the corpus callosum. DLPFC remodeling may reflect synaptic optimization and enhanced integration with hippocampal and contralateral prefrontal networks. Prior work demonstrates that successful episodic retrieval relies on prefrontal regulation of hippocampal reinstatement, which is influenced by thalamic input (68). Together, our findings suggest that changes in microstructural integrity in the ATR_L are associated with better memory performance, potentially via their relationship with cortical thickness changes in the right DLPFC. However, these observations should be interpreted with caution given the small sample size and the fact that all variables in the mediation model reflect change values derived from the same interval, limiting causal inference.

Metabolic improvements after eTRE, including enhanced insulin sensitivity and lower fasting glucose, provide physiological support for the structural changes observed. Insulin signaling promotes NMDA-receptor–dependent synaptic plasticity and dendritic spine remodeling in prefrontal and hippocampal circuits (72, 73). Improved glucose regulation is associated with preserved hippocampal structure and better long-term memory performance (74), thereby providing a supportive metabolic environment for effective prefrontal–hippocampal interactions.

### Limitations

This study has several limitations. First, the sample size was relatively small and the intervention duration was limited to 1 month, which may have reduced statistical power and contributed to only a subset of outcomes reaching statistical significance. Second, the absence of a control group limits causal attribution of the observed pre–post changes in metabolic, cognitive, and neuroimaging measures specifically to the eTRE intervention. Time-related effects and repeated-measure effects (e.g., practice or learning effects on cognitive tasks) cannot be excluded. Third, the study intentionally recruited only young-to-middle-aged male participants to evaluate early intervention potential in a demographic in which cardiometabolic risk factors tend to emerge earlier; however, this design choice limits the generalizability of the findings to females, older adults, and other populations. In addition, the exclusively Asian sample further restricts generalizability across ethnicities. Fourth, potential sources of variability were not fully characterized or controlled, including chronotype differences that may influence meal timing and frequency within a fixed 08:00–16:00 eating window, and baseline dietary habits that may have changed during the intervention. Fifth, while adherence was monitored and participants achieved ≥80% adherence with only minimal deviations, we did not model adherence variability in the analyses. Accordingly, these results should be interpreted with caution. Future studies with larger samples, randomized controlled designs, inclusion of both sexes, more diverse populations, longer follow-up periods, and more comprehensive assessment of chronotype, dietary intake, and adherence are warranted to confirm and extend these findings.

## Conclusion

This study provides multimodal neuroimaging evidence that eTRE was associated with improvements in metabolic status, white-matter integrity, cortical morphology, and cognitive performance in MetS. The identified white matter–cortical–cognitive pathway suggests that the right DLPFC remodeling may be involved in the association between enhanced ATR_L integrity and improved memory performance following 1-month eTRE.

## Data Availability

The datasets presented in this article are not readily available because ethical restrictions imposed by the institutional review board and participant consent terms prohibit the sharing of raw MRI and clinical data. These data contain potentially identifiable information, and public release is not permitted. Requests to access the datasets should be directed to Yarong Wang, wangyr9t@xjtu.edu.cn.
