# Supplementary material for: One-month early time-restricted eating enhances cognition via white matter–cortical pathways in males with metabolic syndrome: evidence from TBSS and SBM analyses
Source: Front Nutr. 2026 Mar 13;13:1753462. doi: 10.3389/fnut.2026.1753462 (PMC13021466; doi:10.3389/fnut.2026.1753462)

Supplementary Material

# CGM-Based Detection of Out-of-Window Ingestion Events in Time-Restricted Eating (TRE)

1. Continuous Glucose and Behavioral Monitoring

All participants were equipped with a continuous glucose monitoring system (CGM) for real-time glucose tracking, and a Huawei Band 8 smart band to monitor sleep and physical activity. Participants were instructed to wear both devices continuously (24 h/day) throughout the 4-week intervention to ensure data completeness and accuracy.

The CGM system was configured to trigger hypoglycemia alerts when interstitial glucose levels fell below 3.9 mmol/L. Across the cohort, hypoglycemia alerts accounted for less than 1% of total recordings, with approximately half of participants exhibiting fewer than 0.1% alerts. Sleep duration and physical activity remained stable across participants, except for one individual who demonstrated greater intra-individual variability without compromising overall data integrity.

2. Detection of Out-of-Window Ingestion Events

2.1 Eating Window and Deviation Criteria

Participants followed a fixed early time-restricted eating (eTRE) schedule from 08:00 to 16:00 daily. Any caloric intake outside this window was considered a protocol deviation.

2.2 Automated Spike Detection Algorithm

An “out-of-window ingestion” event was defined as a glucose excursion meeting all of the following conditions: Amplitude Criterion: a rise of ≥0.8 mmol/L above the preceding baseline; Rate-of-Change Criterion: a rate of increase >0.02 mmol/L/min (≈0.3 mmol/L within 15 minutes).These thresholds were determined from prior literature showing that healthy individuals typically exhibit postprandial excursions of 2.5–3.0 mmol/L^1^ and supported by CGM-based meal detection studies^2,3^, which reported optimal sensitivity for amplitude thresholds between 0.5–1.0 mmol/L and slopes of 0.015–0.03 mmol/L/min.

2.3 Duration and Boundary Tolerance

To reduce false-positive detection due to transient fluctuations, a glucose elevation was considered a valid out-of-window ingestion event only if:
(i) the elevation lasted ≥30 minutes, and
(ii) occurred >30 minutes away from either boundary of the eating window (08:00 or 16:00).

2.4 Day Classification and Adherence Threshold

Any spike fulfilling these criteria and not associated with a self-reported meal or physical activity episode was labeled as an out-of-window ingestion event. A non-adherent day was defined as any day containing ≥1 such event. Participants with ≥80% of days free from out-of-window ingestion were categorized as adherent^4^; others were categorized as non-adherent.

2.5 Calibration and Sensitivity Validation

A 7–14-day pilot calibration phase was conducted before the main intervention to optimize event detection parameters for the specific CGM system. During calibration, CGM-derived glucose traces were matched with participants’ self-reported meal logs to evaluate combinations of amplitude thresholds (0.6, 0.8, 1.0 mmol/L) and rate thresholds (0.015–0.03 mmol/L/min). Receiver operating characteristic (ROC) analysis was used to identify the parameter combination that maximized both sensitivity and specificity. The optimal set was adopted for all subsequent analyses ^3,5^.

2.6 Participant Feedback and Adherence Support

According to the above criteria, all participants achieved ≥80% adherence. A total of 38 out-of-window episodes were detected: 20 associated with physical activity and 18 due to dietary non-adherence.

Each participant was provided with an individualized online platform for continuous data upload, communication, and personalized feedback. The multidisciplinary team (MDT) reviewed daily logs of diet, sleep, and physical activity, offering real-time behavioral and dietary counseling. In addition to adherence support, the research team also responded to hypoglycemia alerts in real time, contacting participants to confirm symptoms and provide individualized dietary guidance when necessary.

Participants completed short daily questionnaires on hunger and emotional state, which facilitated timely psychological and behavioral adjustments. This integrated feedback system helped maintain high adherence and ensured participant privacy throughout the intervention.

**References**

1. Freckmann G, Schauer S, Beltzer A, et al. Continuous glucose profiles in healthy people with fixed meal times and under everyday life conditions. *J Diabetes Sci Technol*. 2024;18(2):407-413. doi:10.1177/19322968221113341

2. Brummer J, Glasbrenner C, Hechenbichler Figueroa S, Koehler K, Höchsmann C. Continuous glucose monitoring for automatic real-time assessment of eating events and nutrition: A scoping review. *Front Nutr*. 2024;10:1308348. doi:10.3389/fnut.2023.1308348

3. Zheng M, Ni B, Kleinberg S. Automated meal detection from continuous glucose monitor data through simulation and explanation. *J Am Med Inform Assoc JAMIA*. 2019;26(12):1592-1599. doi:10.1093/jamia/ocz159

4. Pan G hui, Zhang J qing, Sun Y yan, Shi Y hui, Zhang F rong. Saturation association between serum 25-hydroxyvitamin D levels and mortality in elderly people with hyperlipidemia: A population-based study from the NHANES (2001-2016). *Front Endocrinol*. 2024;15:1382419. doi:10.3389/fendo.2024.1382419

5. Dassau E, Bequette BW, Buckingham BA, Doyle FJ. Detection of a meal using continuous glucose monitoring: Implications for an artificial beta-cell. *Diabetes Care*. 2008;31(2):295-300. doi:10.2337/dc07-1293

# TBSS analysis

Table S1. White matter regions showing increased FA after 1-month eTRE intervention in MetS patients (n = 21)

| White Matter Tract | Peak MNI Coordinates | | | *T* | *p* | Cluster Size |
| --- | --- | --- | --- | --- | --- | --- |
|  | X | Y | Z |  |  |  |
| ATR_L | -7 | -28 | -30 | 3.099 | 0.042* | 19 |
| CST_L | -2 | -29 | -30 | 3.193 | 0.042* | 53 |
| CST_R | 3 | -24 | -34 | 3.423 | 0.043* | 24 |

Note: Values are reported as cluster size, peak Montreal Neurological Institute (MNI) coordinates, peak t value, and FWE-corrected p values. ATR, anterior thalamic radiation; CST, corticospinal tract.

# SBM analysis

Table S2a. Cortical regions showing significant reductions in cortical thickness after 1-month eTRE intervention in MetS patients (n = 21)

| atlas region | brain region | Broadman Division | x | y | z | Cluster-Size | *P* | *T* |
| --- | --- | --- | --- | --- | --- | --- | --- | --- |
| S_front_sup-R | DLPFC-R | BA8/9 | 23 | 10 | 60 | 31 | 0.020* 5.10 | |
| G_front_sup-R |  |  | 22 | 20 | 51 | 104 |  |  |

Note: Values represent cluster size, peak Montreal Neurological Institute (MNI) coordinates, peak *t* values, and FDR-corrected *p* values from SBM analysis.

S_front_sup-R, right superior frontal sulcus; G_front_sup-R, right superior frontal gyrus；DLPFC, dorsolateral prefrontal cortex.

Table S2b. Cortical regions showing significant increases in sulcal depth after 1-month eTRE intervention in MetS patients(n=21).

| atlas region | Broadman Division | x | y | z | Cluster-Size | *P* | *T* |
| --- | --- | --- | --- | --- | --- | --- | --- |
| S_front_inf-L | BA44 | -53 | 30 | 7 | 85 | 0.011* | 4.60 |
| G_front_inf-Triangul-L | BA45 | -46 | 31 | 11 | 96 |  |  |

Note: S_front_inf-L, left inferior frontal sulcus; G_front_inf-Triangul-L, left inferior frontal gyrus, triangular part.

Table S2c. Cortical regions showing significant increases in fractal dimension after 1-month eTRE intervention in MetS patients (n = 21).

| atlas region | brain region | Broadman Division | x | y | z | Cluster-Size | *P* | *T* |
| --- | --- | --- | --- | --- | --- | --- | --- | --- |
| G_front_middle-L | DLPFC-L | BA9 | -13 | 26 | -18 | 36 | 0.047* | 5.09 |
| S_orbital_lateral-L | OFC-L | BA47 | -5 | 26 | -25 | 35 | 0.047* | 4.34 |
| S_orbital_med-olfact-L |  | BA11/BA10 | -39 | 51 | 2 | 38 |  |  |
| G_rectus-L |  | BA10 | -39 | 42 | -3 | 24 |  |  |

Note: Values represent peak Montreal Neurological Institute (MNI) coordinates, cluster size, peak t values, and FDR-corrected p values from SBM analysis. G_front_middle-L, left middle frontal gyrus; S_orbital_lateral-L, left lateral orbital sulcus; S_orbital_med-olfact-L, left medial orbital–olfactory sulcus; G_rectus-L, left rectus gyrus; DLPFC, dorsolateral prefrontal cortex; OFC, orbitofrontal cortex.

# Correlation analysis

Figure S1. Scatter plots showing the correlations between increased fractional anisotropy (FA) in the left anterior thalamic radiation (ATR_L) cluster and metabolic improvements, including reduced fasting glucose (r = −0.513, p = 0.017) and increased QUICKI (r = 0.492, p = 0.024).


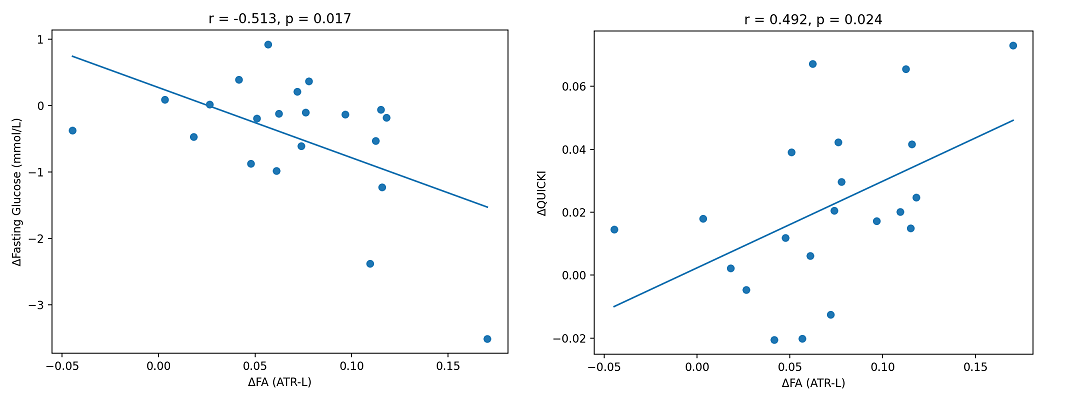


Figure S2. Scatter plots showing the correlations between reductions in cortical thickness in the right DLPFC cluster and improvements in cognitive performance, including better delayed recall (r = −0.505, p = 0.020), a lower TMT B/A Ratio (r = −0.463, p = 0.035), and faster processing speed (s) (r = 0.520, p = 0.016).


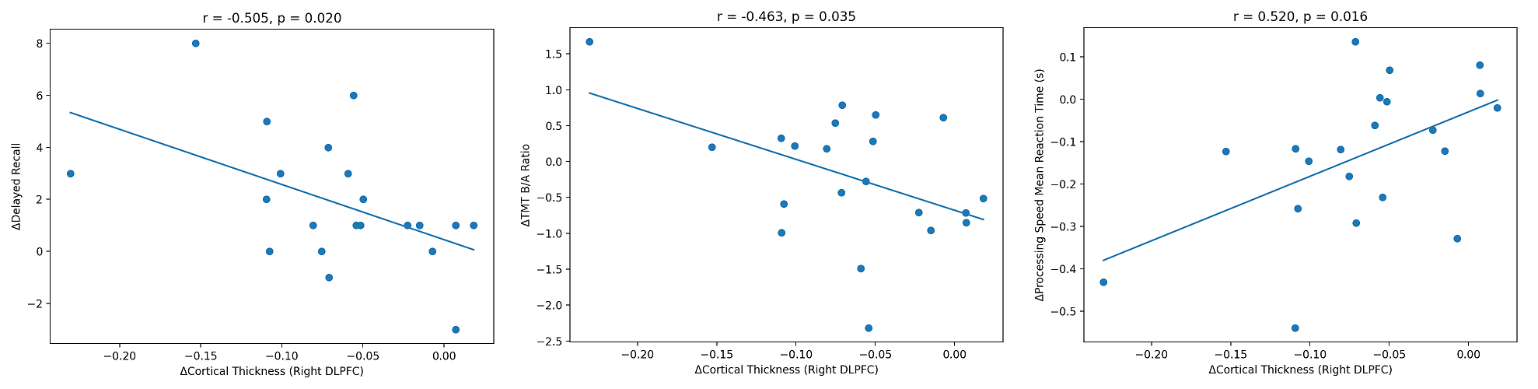

Supplement: Supplementary file 1 [file Table_1.docx]
